# RXR Agonists Enhance Lenalidomide Anti-Myeloma Activity and T Cell Functions while Retaining Glucose-Lowering Effect

**DOI:** 10.3390/cells12151993

**Published:** 2023-08-03

**Authors:** Jian Wu, Xiaobei Wang, Min Zhang, Parker Mathews, Yubin Kang

**Affiliations:** Division of Hematologic Malignancies and Cellular Therapy, Department of Medicine, Duke University Medical Center, Durham, NC 27710, USA; jw731@duke.edu (J.W.); xiaobei.wang@duke.edu (X.W.);

**Keywords:** immunomodulatory drugs, *PPAR*, diabetes, dyslipidemia, CpG island, methylation, metabolomics, treatment response, survival

## Abstract

Retinoid X receptor (*RXR*) heterodimerizes with the *PPAR* nuclear hormone receptor and regulates its downstream events. We investigated the effects of *RXR* agonists (LG100754, bexarotene, AGN194204, and LG101506) on lenalidomide’s anti-myeloma activity, T cell functions, and the level of glucose and lipids in vivo. Genetic overexpression and CRISPR/Cas9 knockout experiments were conducted in multiple myeloma (MM) cell lines and Jurkat T cell lines to determine the roles of *CRBN* in *RXR*-agonist mediated effects. A xenograft mouse model of MM was established to determine the combination effect of LG100754 and lenalidomide. The combination of *RXR* agonists and lenalidomide demonstrated synergistic activity in increasing *CRBN* expression and killing myeloma cells. Mechanistically, the *RXR* agonists reduced the binding of *PPARs* to the *CRBN* promoter, thereby relieving the repressor effect of *PPARs* on *CRBN* transcription. *RXR* agonists downregulated the exhaustion markers and increased the activation markers of Jurkat T cells and primary human T cells. Co-administration of LG100754 and lenalidomide showed enhanced anti-tumor activity in vivo. LG100754 retained its glucose- and lipid-lowering effects. *RXR* agonists demonstrate potential utility in enhancing drug sensitivity and T-cell function in the treatment of myeloma.

## 1. Introduction

Multiple myeloma (MM) is a clonal plasma cell malignancy. It is the second most common hematologic malignancy in the United States [1,2]. Although current anti-myeloma agents, such as immunomodulatory agents, proteasome inhibitors, and monoclonal antibodies, have significantly improved the outcomes of MM patients, MM remains an incurable disease with high rates of drug resistance and relapse [3,4]. There are unmet needs for the development of novel anti-myeloma agents and for understanding the mechanisms of MM’s drug resistance.

Peroxisome proliferator–activator receptors (*PPARs*) belong to the nuclear hormone receptor superfamily of ligand-activated transcription factors. *PPARs* are involved in the regulation of gene expression in response to nutritional and physiological stimuli [5,6]. Moreover, *PPARs* form heterodimers with the retinoid X receptor (*RXR*) to regulate glucose and lipid metabolism [7,8]. The activated ligand-bound *PPAR*s then undergo a conformational change. They subsequently bind to peroxisome proliferator response elements (*PPREs*), thereby leading to transcriptional regulation [9,10]. During this process, additional co-activators or repressors are recruited to create a complex to fine-tune the expression of large gene arrays [11]. Dysfunctional regulation of the expression in these genes is implicated in the development of a range of human diseases, including atherosclerosis, cancer, diabetes, and obesity.

MM occurs largely in elderly. The elderly commonly has a high incidence of comorbidities, including diabetes and dyslipidemia. *PPAR* agonists, such as fibrates and thiazolidinediones, are classes of Food and Drug Administration (FDA)-approved agents used for treating diabetes and dyslipidemia. Our previous research demonstrated that administration of *PPAR* agonists downregulated the expression of cereblon (*CRBN*) by facilitating the binding of *PPAR* to the *CRBN* promoter region and repressing *CRBN* transcription [12]. *CRBN* is a direct target of immunomodulatory agents. Administration of PPAR agonists, such as fenofibrate, GW501516, and troglitazone, resulted in decreased *CRBN* at the mRNA and protein levels [12]. Furthermore, we found that administration of PPAR agonists resulted in the attenuation of the anti-myeloma activity of lenalidomide in vitro and in vivo. These finding led us to explore novel agents that can target the *PPAR* pathway and enhance *CRBN* expression while retaining the beneficial effects of *PPAR* agonists on glucose and lipid metabolism.

*RXRs* are key molecules in the *PPAR* signaling pathway. *RXRs* (RXRα, β, and γ) are master coordinators of cell growth, metabolism, and development [13,14,15]. Among the three subtypes, malfunctioning *RXRα*, which is due to post-translational modification by phosphorylation, is associated with hepatic carcinogenesis [16]. The phosphorylated form of *RXRα* (*p-RXRα*) lost its transactivation activity and interfered with the function of the remaining normal *RXRα* in a dominant-negative manner, thereby promoting the grow of hepatoma cells. Although *RXRs* exists in three isoforms, they do not confer different functions in various *RXR*–*PPAR* complexes [17]. *RXRs* heterodimerize with many different nuclear receptors, including the retinoic acid receptor (*RAR*), the vitamin D receptor (VDR), PPAR, the liver X receptor (LXR), and Nurr77 [18,19]. The RXR/PPAR complex plays a critical role in retinoic acid metabolism, with documented clinical applications in inflammatory disease and certain cancers [20,21,22]. RXR/PPARγ heterodimers reportedly regulate the transcription of genes involved in insulin action, adipocyte differentiation, lipid metabolism, and inflammation [23,24]. Hence, there has been considerable interest in utilizing a combination of ligands for *PPAR* and *RXR* for the prevention and treatment of various types of cancer. However, the significance of the interactions of RXR/PPAR in MM remains unclear; hence, there is a strong need to critically evaluate the specific role of these complexes in MM pathobiology and their responses to current therapeutic strategies.

*RXR* agonists exert their effects through *RXR* homodimers or through permissive heterodimers. LG100754, a novel RXR dimer modulator, is an agonist of RXR/PPARα and RXR/PPARγ heterodimers [25,26,27]. Although LG100754 activates the RXR/PPAR heterodimer, it does not activate TR/RXR, VDR/RXR, or LXR/RXR. At low concentrations, LG100754 acts as a weak agonist of RXR/PPARγ. However, it strongly enhances signaling through the heterodimer in response to PPARγ ligands, including the drug rosiglitazone [27]. LG100754 activates endogenous RXR/PPARγ heterodimer-mediated pathways through inducing adipocyte differentiation of 3T3-L1 cells. Moreover, LG100754 blocks TNF α-mediated inhibition of the insulin receptor (IR) phosphorylation in mature adipocytes. Through this mechanism, LG100754 prevents hyperglycemia in murine diabetes models, thereby reducing insulin resistance [25]. Although the various roles of LG100754 have been identified in different metabolic physiological processes, such as glucose regulation, its function in cancer remains unknown. Bexarotene is an FDA-approved RXR-specific agonist used in the treatment of cutaneous T cell lymphoma (CTCL) [28]. Bexarotene stimulates the formation of RXR and PPARγ heterodimers [29]. The *RXR* agonist, AGN194204, acted through the RXR–PPAR heterodimer and potentiated the anti-tumor effects of PPAR agonists on breast cancer cell lines [30]. LG101506 is the most potent among the selective RXR–PPAR heterodimer activators. Binding to *RXR* results in the selective activation of RXR: PPARα, RXR: PPARγ, and RXR: PPAR δ heterodimers [31]. LG101506 lowers blood glucose levels in genetically predisposed type II diabetic mice [32]. The effects of these RXR agonists on anti-myeloma activity either alone or in combination with immunomodulatory drugs (IMiDs) are unknown.

In this study, we hypothesized that the *RXR* agonists could enhance the sensitivity of myeloma cells to lenalidomide through their heterodimer formalization with PPAR, while maintaining the regulation of blood glucose and lipid levels. We first determined the effects of *RXR* agonists alone and in combination with lenalidomide on anti-myeloma activities in vitro and in vivo in a myeloma xenograft mouse model. Then, mechanistic studies were performed. In addition, the regulatory effects of RXR agonists on glucose and lipid homeostasis and on T cell function were evaluated.

## 2. Materials and Methods

### 2.1. Cell Lines

MM1R, RPMI8226, NCIH929, and U266 MM cell lines were used in this study. These cell lines were purchased from ATCC and authenticated periodically using short tandem repeat profiling. All the cell lines were cultured at 37 °C under 5% CO_2_ in Roswell Park Memorial Institute 1640 medium supplemented with 2 mM GlutaMAX and 10% fetal calf serum (Mediatech, Hsinchu, Taiwan).

### 2.2. Clustered Regularly Interspaced Short Palindromic Repeats (CRISPR)/CRISPR-Associated Protein 9 (Cas9) Genomic Editing

CRBN-knockout using the CRISPR/Cas9 technique was performed as previously described. Briefly, single guide RNAs (gRNAs) targeting the human *CRBN* gene, 5′-CAGGACGCTGCGCACAACAT-3′, and 5′-CGCACCATACTGACTTCTTG-3′ were obtained from Integrated DNA Technologies (Coralville, IA, USA). The gRNA fragment was amplified via a polymerase chain reaction (PCR) prior to use for transfection, as described previously [33]. The cells were transfected with a ribonucleoprotein (RNP) complex using an RNAiMAX kit (Cat# 13778150; Thermo Fisher Scientific (Waltham, MA, USA)). Two pairs of primers were used to verify the successful knockout of *CRBN*: pair 1, forward: 5′-TCCTTTGCGGGTAAACAGAC-3′ and reverse: 5′-GGTTGGAATCCTGACTCTGC-3′; and pair 2, forward: 5′-TGGCACAATCTCAGCTCACT-3′ and reverse: 5′-ACCACTGCAATTACCCATGA-3′. T7E1 digestion followed by electrophoresis at 100 V for 1.5–2 h in 3% (*w*/*v*) agarose gels was used to detect and visualize the knockout results [34].

### 2.3. CRBN Firefly Luciferase Reporter System

To generate a firefly luciferase reporter plasmid driven by the *CRBN* promoter, a human *CRBN* promoter was subcloned into a pGL3-basic vector containing the firefly luciferase gene (Promega (Fitchburg, WI, USA), GenBank accession number U47295). The *CRBN* promoter sequences, 2000 bp upstream of the *CRBN* start site, were obtained from the NCBI database. Potential *PPAR* binding sites in the *CRBN* promoter region were identified using the JASPAR database (http://jaspar.genereg.net/, accessed on 1 September 2021) [35]. The *PPARα* binding site was identified as follows: 5′-TTGAGCTCTTCCCTACTC-3′, *PPARβ/δ* binding site: 5′-GCGATCTTCAACCTCA-3′, and *PPARγ* binding site: 5′-TCCCCTGTCACCTTC-3′. The *CRBN* promoter regions containing various *PPAR* binding sites were PCR-amplified with primers as follows: for the *CRBN* promoter with a *PPARα* binding site, forward: 5′-AGATAAGGGGCTGAGCTTCC-3′ and reverse: 5′-ATGTTTGACTCATTTGGTTGAAGA-3′; for the *CRBN* promoter with a *PPARγ* binding site, forward: 5′-CCAACTTAAAGGCGAACCAC-3′ and reverse: 5′-GGAACTCTTGATGTAGCTTTAATGG-3′; and for the CRBN promoter with a *PPARβ/δ* binding site, forward: 5′-AACTATAAATAAGCCAAGGTTTTTCTC-3′ and reverse: 5′-TCTTTTGGCCTCATTATTCAAA-3′. The pGL3-basic firefly luciferase reporter vector was used as the negative control.

### 2.4. Methylation Specific PCR (MSP)

The genomic DNA was extracted from the cultured cells and subjected to MS-PCR analysis as described [36]. Briefly, U266 and MM1.R cells were treated with DMSO control, 8 μM LG100754, 4 μM Bexarotere, 4 μM AGN194204, or 4μM LG101506 for 48 h. The cells were then harvested, and the DNA was isolated using a tissue/cell genomic DNA isolation kit with the Wizard DNA clean-up kit (Promega, Fitchburg, WI, USA). The DNA was then treated with bisulfite according to the manufacturer’s instructions (EZ DNA Methylation Kit, Zymo Research, Irvine, CA, USA), and amplified by PCR with two pairs of specific primers that recognize the methylated (M) and the unmethylated (U) CpG sites in the *CRBN* promoter. The primers were designed using the MethPrimer program (http://www.urogene.org/methprimer/ accessed on 23 June 2023). The primer pair for the methylated form (129 bp) was as follows: forward: GAATAAAGTGAGGGTTTTGTAGC; reverse: ACCTAAAAATAATAACCTAAACGAA. The primer pair for the unmethylated form (131 bp) was the following: forward: TGGAATAAAGTGAGGGTTTTGTAGT; reverse: ACCTAAAAATAATAACCTAAACAAA. The PCR amplification conditions were 95 °C for 5 min; 40 cycles at 95 °C for 45 s, 60 °C for 45 s, 72 °C for 45 s, and, finally, 10 min at 72 °C. The PCR products were visualized in Gene Genius (Syngene, Cambridge, UK) by ethidium bromide staining in 2% agarose gels.

### 2.5. Lentiviral Gene Transduction

For lentiviral production, HEK293 cells were transfected with triple plasmids: lentiviral vector plasmid, packing plasmids PMD2.G, and psPAX2 (Addgene, plasmid#12259 and 12260). Forty-eight hours later, the cell supernatant was collected and concentrated using Lenti-X Concentrator (Takara Bio, San Jose, CA, USA) per the manufacturer’s instruction. For lentiviral gene transduction, the cells were seeded on a six-well plate (Corning, New York, NY, USA) and transduced with lentivirus with polybrene (8 μg/mL). The cells were treated with puromycin (Sigma, Burlington, MA, USA) for 7 days to select the transduced cells.

### 2.6. Co-Immunoprecipitation

Co-immunoprecipitation (co-IP) was performed according to the manufacturer’s instructions (Pierce Co-Immunoprecipitation Kit, Thermo Scientific, Waltham, MA, USA, #26149). Antibodies against PPARα (Abcam, #ab227074), PPARγ (Proteintech, 16643-I-AP), and PPARδ (#74076, Cell signaling Technology, Danvers, MA, USA) were used.

### 2.7. Reagents and Antibodies

The PPARα antibody was obtained from Santa Cruz Biotechnology, Dallas, TX, USA (Cat# SC-398394). The PPARβ/δ (NBP2-22468) and PPARγ (NBP2-22106) antibodies were obtained from Novus Biologicals, LLC. (Littleton, CO, USA). The IKZF1 antibody was purchased from Santa Cruz Biotechnology, Inc. (Dallas, TX, USA). The IKZF3 antibody was purchased from Novus. The CRBN antibody was obtained from Sigma-Aldrich (St. Louis, MO, USA). The Caspase 3 antibody, EZH2 antibody (#4905S), and H3K27me3 antibody (#9733T) were purchased from Cell Signaling Technology (Danvers, MA, USA).

### 2.8. Thiazolyl Blue Tetrazolium Bromide (MTT) Cell Proliferation Assay

MM cells were plated in triplicate in a 96-well plate (5 × 10^4^ cells/well at a final volume of 100 μL) and treated with various concentrations of lenalidomide and/or RXR agonists for the indicated durations. To measure the cell proliferation, 20 μL of the combined MTS/PMS solution (5 mg/mL MTT) was added to each well and incubated for 3–4 h at 37 °C in a 5% CO_2_ incubator. The absorbance at 490 nm was measured using an enzyme-linked immunosorbent assay plate reader (VERSAmax, Molecular Devices, San Jose, CA, USA).

### 2.9. Western Blot Analysis

The MM cells were harvested, washed with phosphate-buffered saline (PBS), and resuspended in a lysis buffer containing 50 mM Tris-HCl (pH 7.4), 150 mM NaCl, 1 mM EDTA, 1% Triton X-100, 1% sodium deoxycholate, and 0.1% sodium dodecyl sulfate (SDS). The cells were lysed via brief sonication and then spun down to remove the cell debris. The total protein in the cell lysates was quantified using a Dc protein estimation kit (Bio-Rad, Hercules, CA, USA) with bovine serum albumin (BSA) as a standard. The cell lysates were loaded and run on SDS-polyacrylamide gel electrophoresis. The proteins were transferred onto a nitrocellulose membrane. The membrane was blocked with 5% BSA in tris-buffered saline containing 0.05% Tween 20 (TBST) and incubated with primary antibodies against PPARα (ab227074; Abcam, Waltham, MA, USA), PPARβ/δ (A5656; Abclonal, Woburn, MA, USA), PPARγ (16643-1-AP; Thermo Fisher, Waltham, MA, USA), IKZF1 (NBP1-98314; Novus Biologicals), and IKZF3 (NBP2-46048; Novus Biologicals, Littleton, CO, USA) in TBST containing 5% BSA overnight at 4 °C with gentle rocking. The membrane was then probed with an HRP-conjugated secondary antibody and developed using a Pierce ECL substrate.

### 2.10. Flow Cytometry for the Measurement of T Cell Activation and Exhaustion

The Jurkat T cells and primary patient T cells were characterized for markers of activation and exhaustion using flow cytometry. The cells were washed with PBS and centrifuged with 500× *g* for 5 min. The supernatant was discarded, and the cells were stained with 100 µL FACs buffer-contained antibody for 15 min at 4 °C. The cells were washed with 500 µL PBS and intracellular staining for the IFN-γ, and Granzyme was performed using an eBioscience Foxp3 Transcription Factor Staining Buffer Set (Invitrogen, Carlsbad, CA, USA, cat#00-5523-00) according to the manufacturer’s instructions. Briefly, 500 µL fixation buffer was used to incubate the samples overnight at 4 °C and 1 mL permeabilization buffer was added to the samples. The samples were washed, resuspended in 100 µL permeabilization buffer containing 2 µL antibody, and incubated at 4 °C for 30 min. The samples were washed again and fixed with 0.5% paraformaldehyde (PFA, Gibco, Grand Island, NY, USA), and were then ready for the analysis by flow cytometry.

The following antibodies were used for cell surface or intracellular stains: granzyme-B (Cat#561998, BD Biosciences, San Jose, CA, USA); anti-IFN-γ APC (Biolegend, San Diego, CA, USA, Cat#502512); CD69 PE-Cy7(BD Biosciences, San Jose, CA, USA, Cat#310911); CD223 (LAG-3) (Biolegend, San Diego, CA, USA, Cat#369211); CD279 (PD-1) (Biolegend, San Diego, CA, USA, Cat#329905); CD366 (Tim3) (Biolegend, San Diego, CA, USA, Cat#345013); CD152 (CTLA-4) (Biolegend, San Diego, CA, USA, Cat#369633); TIGIT (Biolegend, San Diego, CA, USA, Cat#372717); and CD3 (Cat#561806, BD Biosciences, San Jose, CA, USA).

### 2.11. Chromatin Immunoprecipitation (ChIP) Assay

ChIP assays were performed as previously described. Briefly, MM cells were collected, cross-linked with 1% formaldehyde, and centrifuged. The resulting pellets were sonicated, and the chromatin solution was precleared with 30 μL of ChIP-grade protein G magnetic beads (#9006; Cell Signaling Technology, Danvers, MA, USA). The soluble fraction was collected, and the chromatin beads were incubated with positive control histone H3 rabbit (#4620; Cell Signaling), normal rabbit IgG (#2729; Cell Signaling), anti-PPARα (ab227074; Abcam), anti-PPARγ (16643-1-AP; Proteintech Rosemont, IL, USA), and anti-FLAG M2 (F1804; Sigma; for PPARβ/δ with a FLAG tag) antibodies at 4 °C overnight. The ChIP-enriched DNA was analyzed by quantitative PCR using the CRBN promoter primers as follows: forward: 5′-TCCCCTGTCACCTTCAAAAC-3′ and reverse: 5′-TGCCTTGTGAGTCTGACACC-3′. The enrichment of the CRBN promoter regions was assessed relative to the input DNA, followed by normalization to the respective control IgG values. Percent input = 4% × 2(C[T] 4% input sample- − C[T] IP sample).

### 2.12. Confocal Microscopy Examination of CRBN in MM Cell Lines

A Confocal examination of *CRBN* was performed as described. Glass slides were first coated with 10 μg/mL of fibronectin (catalog 341635; Sigma-Aldrich, St. Louis, MO, USA). The MM cells were then added and remained on the slides for 1 h at 37 °C. The cells were subsequently fixed with 4% formaldehyde in PBS for 15 min at room temperature, blocked with cell culture medium containing 10% fetal bovine serum, and incubated overnight at 4 °C with the CRBN antibody (PA598707; Thermo Fisher Scientific, Waltham, MA, USA). The slides were then washed thrice with PBS, stained with Alexa Fluor 594 goat anti-rabbit antibody (R37117; Thermo Fisher Scientific) for 1 h, labeled with 4′,6-diamidino-2-phenylindole dihydrochloride (DAPI; 4083; Cell Signaling) for 5 min, and mounted with the antifade mounting medium (Vector, H-1000). Images were acquired using a confocal laser-scanning microscope (Leica SP5 inverted confocal microscope). Sequential scanning of the different channels was performed at a resolution of 512 × 512 pixels. The brightness was optimized and applied to the entire image.

### 2.13. In Vivo Glucose and Lipid Measurement

C57Bl/6 mice were given LG100754 (5 mg/kg intraperitoneally). Blood samples were collected at the time-points indicated. The blood glucose level was measured by an AimStrip Plus Blood Glucose Testing System. The total lipids were determined with quantitative enzymatic assays using the lipid quantification kit STA-613 (Cell Biolabs, INC., San Diego, CA, USA, Cat#101420224). The procedures were performed in accordance with the protocols provided by the manufactures.

### 2.14. Myeloma Xenograft Mouse Model

Immunodeficient (SCID) mice (male, 6–8 weeks old; OCI) were injected subcutaneously with 1 × 10^7^ MM1.R in the lower right flank. When tumors were palpable (approximately 21 days after injection), the mice were randomly assigned into 4 groups (*n* = 7 in each group), receiving an intraperitoneal injection with 10 mg/kg/day [37,38] lenalidomide or in combination with LG100754 5 mg/kg three times a week. The tumors were measured with external calipers every 3 days, and the tumor volume (in mm^3^) was calculated using the following standard formula: V = 0.5a × b^2^, where “a” and “b” are the long and short diameters of the tumor, respectively. Animal survival was monitored from the first day of tumor injection until death. The mice were sacrificed when they reached humane endpoints, such as their tumors reaching 1.5 cm in diameter or in the event of paralysis or a major compromise in their quality of life, to prevent unexpected suffering [39]. All the studies involving vertebrate animal models were conducted in accordance with the approved Duke University Institutional Animal Care and Use protocols.

### 2.15. Statistical Analysis

Statistical analyses were conducted in GraphPad Prism v9 (Dotmatics, Boston, MA, USA). The values represent the mean ± standard error of the mean, unless stated otherwise. Graphical displays were used to determine the appropriate transformations, and in some cases, the data were analyzed on a log scale to adhere to the assumptions of the parametric tests. Comparisons of the mean expression across the groups were performed using two-sample *t*-tests. Based on the distribution within the groups, we used a t-test with equal or unequal variances. For all the comparisons, an alpha level of 0.05 was used to denote significance.

## 3. Results

### 3.1. Synergistic Anti-Myeloma Activity of Lenalidomide and RXR Agonists In Vitro

**Figure 1:**
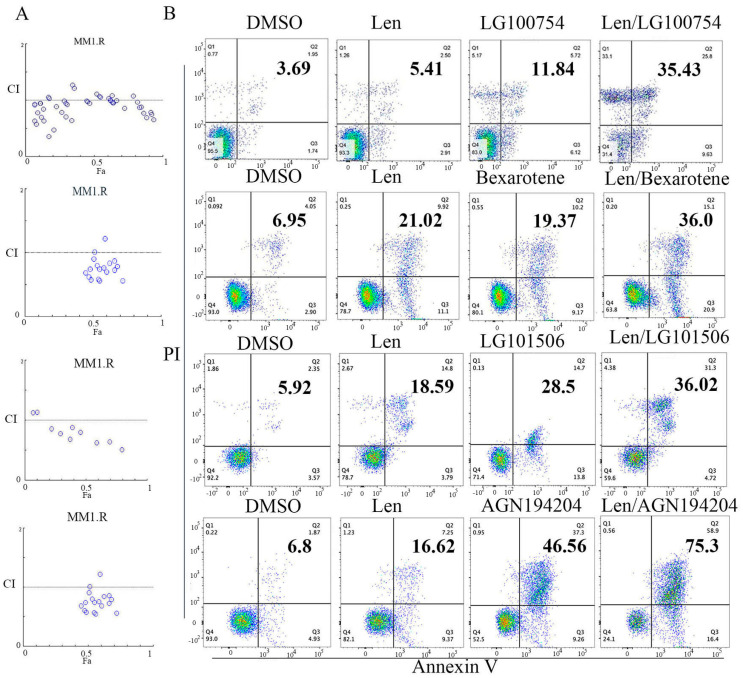
Synergistic suppression of MM cell proliferation using lenalidomide and *RXR* agonists. (**A**) MM1.R were treated with various combinations of different concentrations of *RXR* agonist and lenalidomide for 48 h, and combination index (CI) values were identified using CompuSyn software 1.0 (ComboSyn, Inc (Paramus, NJ, USA)). Fa-CI plots were generated using non-constant ration combination data for each of the drug combinations. Each circles presents one drug combinations. (**B**) Lenalidomide (10 µM), LG100754 (8 µM), bexarotene (4 µM), AGN194204 (4 µM), and LG101506 (4 µM) were used in MM1.R for 48 h; cell apoptosis was measured using Annexin V/PI staining assay.

We examined the anti-myeloma activity of *RXR* agonists alone and in combination with lenalidomide. MM cell viability and apoptosis were first examined. Four RXR agonists (LG100754, bexarotene, AGN194204, and LG101506) were tested on U266 and MM1.R cell lines. We first determined the dose response curves individually and obtained the IC50 value for each of them (Supplementary Figure S1). We then selected a range of drug combination concentrations to evaluate the inhibition of growth with the drug combination of *RXR* agonist and lenalidomide (Figure 1). Compusyn software was used to predict the additive and synergistic effects arising from combinations of multiple drugs with independent mechanisms of action using the combination index theorem [40]. *RXR* agonists (LG100754, bexarotene, AGN194204, or LG101506) demonstrated single agent anti-myeloma activity with an IC50 ranging from 4.5 μM to 20 μM (Supplementary Figure S1). After co-treatment with lenalidomide and the *RXR* agonist, the survival of the MM cells was significantly reduced in comparison with cells that were treated with lenalidomide or the *RXR* agonist alone. Almost all the combination index values were <1. In certain combinations, the combination index (CI) value was below 0.5, suggesting that the growth inhibition effect of lenalidomide and RXR agonists in particular cancer cells is synergistic (Figure 1A and Figure S2). Based on the multiple dose combination strategies, LG100754 (8 µM) with 10 µM lenalidomide and bexarotene, AGN194204, and LG101506 (4 µM) with 8 µM lenalidomide caused about a 50% decrease in cell viability. Moreover, the MM cells exhibited a marked increase in apoptosis when the *RXR* agonists were combined with lenalidomide (Figure 1B). In summary, the cytotoxicity increased significantly when treated with combined lenalidomide and *RXR* agonists compared to lenalidomide or *RXR* agonists alone.

### 3.2. Increased CRBN Expression Correlates with IMiD and RXR Agonist Synergism

**Figure 2:**
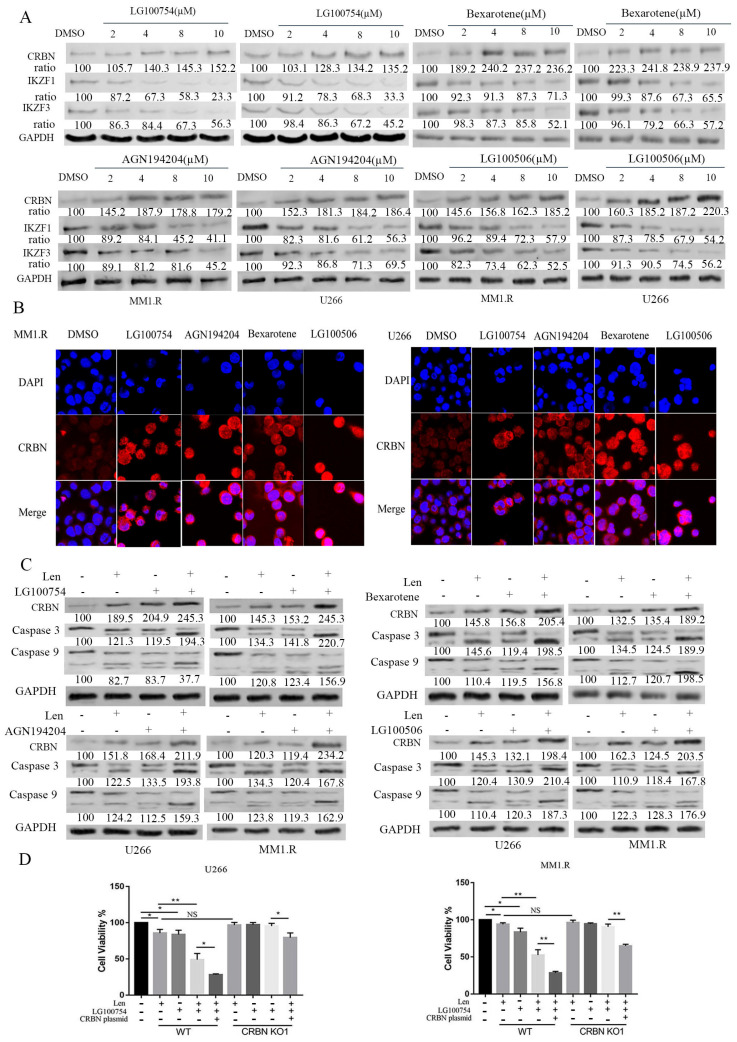
Increased *CRBN* expression is associated with synergistic effect of lenalidomide and RXR agonists. (**A**) U266 and MM1.R were treated with indicated concentrations of RXR agonists for 48 h. Protein lysate was subjected to Western blot with indicated antibodies. (**B**) Representative images of CRBN in MM cells treated with DMSO or 8 µM LG100754, 4 µM bexarotene, 4 µM AGN194204, and 4 µM LG101506 for 48 h. Bar graphs display the results of the mean intensity of CRBN immunofluorescence of two groups. (**C**) U266 and MM1.R were treated with 10 µM lenalidomide and RXR agonists at concentrations indicated in (**B**) for 48 h. CRBN, caspase 3, and caspase 9 expression was measured by Western blot. (**D**) MM1.R and U266 cells were transduced with CRBN-specific CRISP/cas9 knockout vector for 24 h. Cells were then treated with lenalidomide alone, LG100754 alone, or in combination for additional 48 h with or without transduction of CRBN overexpressing plasmid. Cell viability was measured by MTT assay. Results are presented as mean ± SD from at least three separate experiments. NS: not statistically significant; *: *p* < 0.05; **: *p* < 0.01.

*CRBN* is a direct target of immunomodulatory agents and plays a critical role in IMiD-mediated anti-myeloma activity. We thus determined the effect of *RXR* agonists on the expression of *CRBN* in MM. We treated U266 and MM1.R with increasing concentrations of *RXR* agonists and examined their *CRBN* expression after 48 h. U266 and MM1.R showed an appreciable dose response. There was a high induction of *CRBN* at 8 µM of LG100754 and at 4 µM of bexarotene, AGN194204, and LG101506 (Figure 2A). Consistent with the finding, an immunofluorescence analysis showed that the expression of *CRBN* was significantly increased after treatment with the *RXR* agonist at the indicated concentrations (Figure 2B). *IKZF1* and *IKZF3* are transcription factors important in MM cell survival. *CRBN* induces ubiquitination and degradation of *IKZF1* and *IKZF3*. *RXR* agonists induce *CRBN* upregulation and reduce *IKZF1* and *IKZF3* expression (Figure 2A). To further study the synergic effect of the combination of lenalidomide and *RXR* agonists, U266 and MM1.R cell lines were co-treated with lenalidomide and *RXR* agonists at the indicated concentrations. *CRBN* expression was then measured (Figure 2C). In the presence of the *RXR* agonists, the lenalidomide treatment further increased the *CRBN* expression and dramatically promoted the cleavage of caspase 3 and caspase 9, indicative of the induction of apoptosis (Figure 2C). These data show that *RXR* agonists can increase *CRBN* expression alone and can also act synergistically with lenalidomide to further increase *CRBN* expression in MM.

To confirm whether *RXR* agonists synergistically enhance the anti-myeloma activity of lenalidomide through altering *CRBN* expression, we genetically overexpressed *CRBN* in the MM cell lines. The cells were then treated with lenalidomide in the presence or absence of LG100754. Compared with MM cells transduced with an empty vector control, MM cells overexpressing *CRBN* showed increased sensitivity to lenalidomide. Additionally, *CRBN* overexpression enhanced lenalidomide’s anti-myeloma activity in the presence of LG100754 (Figure 2D).

To further validate the role of *CRBN* in lenalidomide and *RXR* agonist-mediated anti-myeloma effects, we used CRISPR/Cas9 technology to knock out the expression of CRBN in the U266 and MM1.R cell lines. The deletion of *CRBN* was confirmed by Western blot (Supplementary Figure S5). The knockout of *CRBN* rendered the MM cells resistant to treatment with lenalidomide, LG100754, and the combination of lenalidomide and LG100754. The transduction of CRBN overexpressing plasmid partially restored the apoptotic induction of lenalidomide and LG100754 (Figure 2D and Figure S5). These data demonstrate the critical role of *CRBN* in potentiating the synergistic anti-myeloma activity of LG100754 and lenalidomide.

### 3.3. RXR Agonists Attenuate the Binding Effect of PPARs on the CRBN Promoter Region in MM Cell Lines

**Figure 3:**
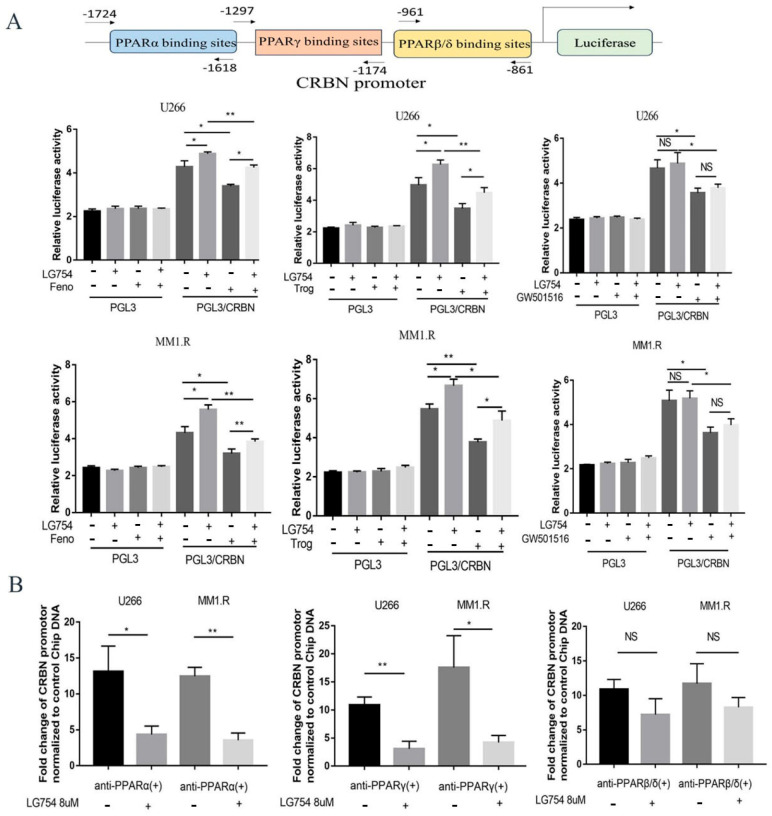
LG100754 attenuates the binding effect of PPARα and PPAR γ on the CRBN promoter area. (**A**) U266 and MM1.R were transfected with CRBN/PGL3 firefly luciferase reported vector construct, then co-treated with PPARs agonist with LG100754 for 48 h, and luciferase bio-luminate activity was measured. (**B**) Bar graphs show qRT-PCR data using immunoprecipitated DNA obtained from ChIP with anti-CRBN or anti-IgG (negative control) antibodies; error bars represent SD. Results are presented as mean ± SD from at least three separate experiments. NS: not statistically significant; *: *p* < 0.05; **: *p* < 0.01.

We wanted to determine the molecular mechanism through which *RXR* agonists enhance *CRBN* expression. Our previous study demonstrated that *PPARs* negatively regulate *CRBN* transcription activity through binding to the *CRBN* promoter region and repressing *CRBN* transcription [12]. The *PPARs* heterodimerized with *RXRs*, translocated to the nucleus, and bound to PPREs to regulate gene expression. We thus hypothesized that *RXR* agonists could affect *PPAR* binding to the *CRBN* promoter region and reverse PPAR’s repressive effects on *CRBN* transcription. To test this hypothesis, we subcloned the *CRBN* promoter into the PGL3 firefly luciferase reporter vector and transfected the construct into U266 and MM1.R cells. The transduced MM cells were then treated with *RXR* agonists (LG100754 or bexarotene) with or without *PPAR* agonists (PPARα agonist fenofibrate, PPARγ agonist troglitazone, or PPARβ/δ agonist GW501516). As shown in Figure 3A, administration of LG100754 increased the *PPARα* and *PPARγ* promoter activities but not the *PPARβ/δ* promoter activity. Furthermore, LG100754 reversed the inhibitory effects of fenofibrate and troglitazone on the activities of the *CRBN* promoter but had no effect on GW501516. These findings are consistent with the function of LG100754, which stabilizes the RXR: PPARα and RXR: PPARγ heterodimers but not PPARβ/δ. For bexarotene, a similar phenotype was only observed for the PPARγ promoter and for PPARγ agonist troglitazone (Figure 3A and Figure S3).

We next determined if the *RXR* agonists affected the binding of the *PPARs* to the *CRBN* promoter. Chromatin immunoprecipitation (ChIP) assay with qPCR was performed for the LG100754-, bexarotene-, AGN194204-, and LG101506-treated MM cells. The binding of PPARs to the *CRBN* promoter was subsequently measured. LG100754 reduced the binding of *PPARα* and *PPARγ*, but not the binding of *PPARβ/δ* to the *CRBN* promoter. As expected, bexarotene only affected the binding of *PPARγ*, but not those of *PPARα* or *PPARβ/δ* to the *CRBN* promoter (Figure 3B and Figure S4).

### 3.4. RXR Agonists Facilitated DNA Demethylation in the CpG Islands within the CRBN Promoter

**Figure 4:**
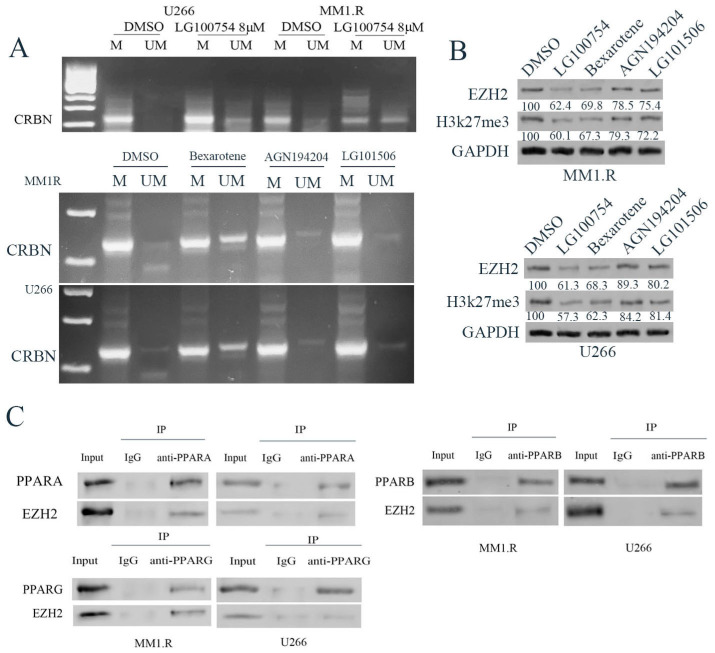
LG100754 changes the methylation pattern of CpG island in *CRBN* promoter region. (**A**) The productions from the methylation-specific PCR run on 2% agarose gels. (**B**) U266 and MM1.R were treated with 8 µM LG100754, 4 µM Bexarotene, 4 µM AGN194204, and 4 µM LG101506 for 48 h. Protein lysate was subjected to Western blot with indicated antibodies. (**C**) Co-IP assay between *PPAR* with *EZH2* was conducted with the indicated antibody in U266 and MM1.R.

DNA methylation is one of the epigenetic events that plays important roles in transcriptional repression, genomic imprinting, and genomic stability [41,42]. Our previous study showed that PPAR agonists induced DNA methylation in the CpG islands of the *CRBN* promoter region [43]. Here, to further analyze the mechanism underlying the enhanced effects of *RXR* agonists on *CRBN* transcription, we performed methylation-specific PCR (MSP) to evaluate the methylation or demethylation status of the CpG island in the *CRBN* promoter region. We treated U266 and MM1.R cells with LG100754, bexarotene, AGN194204, or LG101506. U266 and MM1.R cells are typically resistant to lenalidomide, and they had a lower level of *CRBN* expression (Figure 2). Moreover, they are completely methylated in the CpG islands of the *CRBN* promoter region (Figure 4A). Treatment with LG100754 and bexarotene resulted in significant demethylation of the CpG islands of the *CRBN* promoter region. AGN194204 and LG101506 are less effective in inducing DNA demethylation of the CpG islands (Figure 4A and Figure S6).

Enhancer of zeste homolog 2 (*EZH2*), a histone methyltransferase (*HMT*), induces the trimethylation of histone *H3 lysine 27* (H3K27me3). It is critical in the DNA methylation process [44,45]. We determined the effects of RXR agonists on EZH2 and H3k27me3. U266 and MM1.R were treated with 8 µM of LG100754, 4 µM of bexarotene, 4 µM of AGN194204, or 4 µM of LG101506 for 48 h. The total protein was used for Western blotting. We found that only LG100754 and bexarotene resulted in a decrease in the levels of *EZH2* and *H3K27me3* (Figure 4B). These results are consistent with our MSP data.

We next performed co-IP, followed by Western blotting to determine how the PPAR: RXR heterodimer interacted with *EZH2*. We found that *PPARα*, *PPARβ/δ*, and *PPARγ* form complexes with *EZH2*. Moreover, they interacted with *EZH2* directly (Figure 4C). On the other hand, direct protein interaction between *RXR* with *EZH2* was not detected in our study (Supplementary Figure S7). These data suggest that *RXR* agonists affected the *CRBN* expression, likely indirectly. The effect was most likely produced through modulation of the interaction and binding between *PPARs* and the *CRBN* promoter, including the DNA methylation status.

### 3.5. LG100754 Improved Markers of T-Cell Activity and Suppressed T-Cell Exhaustion Markers

**Figure 5:**
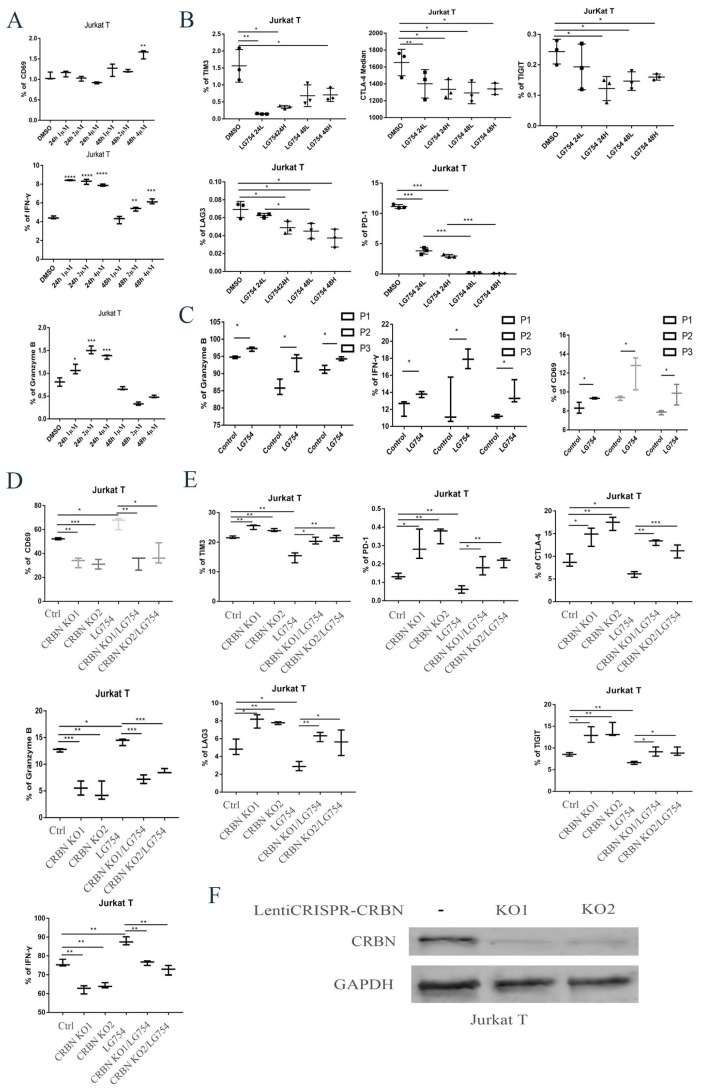
Treatment with RXR agonists resulted in increased T cell activity and decreased checkpoint markers. (**A**,**B**) Jurkat T cell was treated using multiple concentrations (1 µM, 2 µM, and 4 µM) in 24 h and 48 h. *CD69*, *IFN-γ*, *Granzyme B*, and T cell checkpoints, such as *TIM3*, *CTLA-4*, *TIGIT*, *LAG3*, and *PD-1*. Cell were measured using flow cytometry. (**C**) Primary human T cells were isolated from the bone marrow of three myeloma patients (P1, P2, P3), then treated with 2 µM LG100754 for 24 h. *CD69*, *IFN-γ*, *and Granzyme B* were measured using flow cytometry. (**D**,**E**) *CRBN* knockout reversed the effect of LG100754 on Jurkat T cell. Jurkat T cell was treated with 2 µM LG100754 for 24 h in *CRBN*-KO and wild-type cells. *CD69*, *IFN-γ*, *Granzyme B,* and T cell checkpoints (such as *TIM3*, *CTLA-4*, *TIGIT*, *LAG3*, and *PD-1*) were measured using flow cytometry. (**F**) The protein lysates acquired from CRBN knockout Jurkat T cells were analyzed by Western blotting using indicated antibodies. *: *p* < 0.05; **: *p* < 0.01; ***: *p* < 0.001; ****: *p* < 0.0001.

The tumor microenvironment (TME) is a key mediator of tumor development and progression. It also plays an important role in mediating the clinical response to therapy [46,47,48]. T cell activation and expansion in the TME are critical for antitumor immunity [49]. However, anergy, exhaustion, and active suppression can occur in the T cells of MM patients [50,51]. Because *CRBN* was critical in T cell co-stimulation [52], we wanted to determine what the effects of *RXR* agonists were on T cell activation and exhaustion. Jurkat T cells were treated with LG100754 at 1 µM, 2 µM, and 4 µM for 24 and 48 h. *CD69*, *IFN-γ*, *granzyme B*, and T cell checkpoints (*TIM3*, *CTLA-4*, *TIGIT*, *LAG3*, and *PD-1*) were assessed. The cells were then measured using flow cytometry (Figure 5A,B). Our data suggest that LG100754 resulted in significant enhancement of the T cell activation markers (*CD69*, *IFN-γ*, and *granzyme B*) and attenuated T cell exhaustion markers, such as *TIM3*, *CTLA-4*, *TIGIT*, *LAG3*, and *PD-1*.

To evaluate whether LG100754 had enhancing effects on the primary T cells, primary human T cells were isolated from the bone marrow aspirate of patients with MM. The primary human T cells were then treated with 2 µM of LG100754 for 24 h (Figure 5C). This was used to evaluate the effect of LG100754 on T cell’s function. Consistent with the data on Jurkat T cells, LG100754 resulted in an increase in T cell activation markers. The percentage of cells expressing *CD69*, *IFN-γ*, and *granzyme B* was significantly higher with the LG100754 treatment in all three primary T cell samples.

To further verify that the effects of LG100754 were mediated by *CRBN*, we performed a CRISPR/Cas9 assay to knockout *CRBN* in the Jurkat T cells (Figure 5D–F). The *CRBN* knockout reversed the percentage expressing the checkpoint marker above in the presence of LG100754. These findings show that LG100754 induces T cell response by enhancing the efficiency of T cell activation and by protecting T cells from exhaustion in a *CRBN*-dependent mechanism.

### 3.6. LG100754 Enhanced Lenalidomide’s Anti-Myeloma Activity in a Preclinical Model of MM

**Figure 6:**
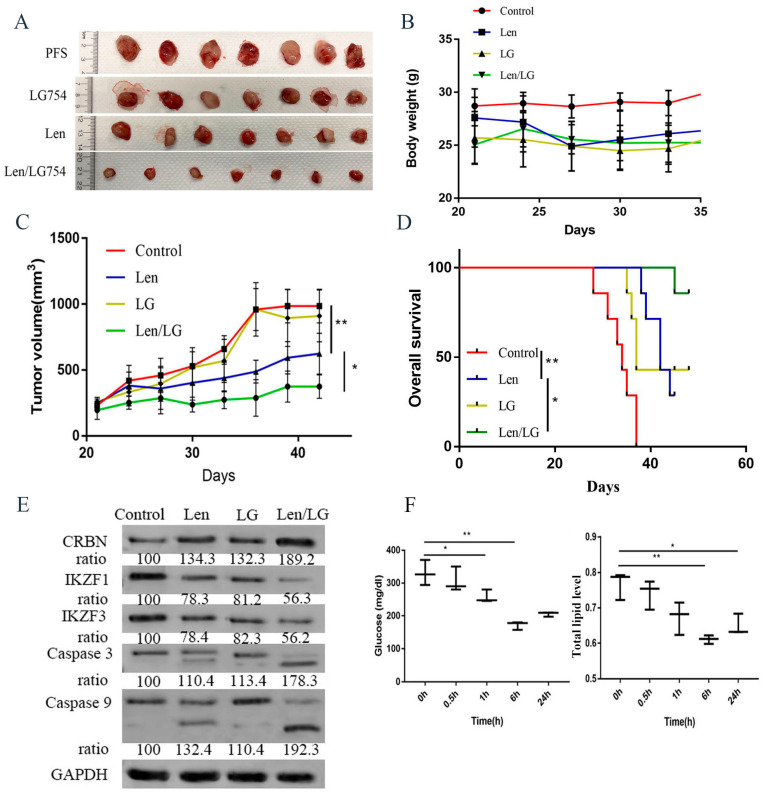
LG100754 attenuates tumor growth and prolongs survival of mice injected with lenalidomide-resistant human MM cells. (**A**) Representative images of SCID mice with subcutaneous MM tumor. (**B**) Body weight was measured every 3 days and presented as means ± SD. (**C**) LG100754 enhanced lenalidomide-induced attenuations of tumor growth in severe combined immunodeficient mice. (**D**) Overall survival was evaluated using Kaplan–Meier curve and long-rank analysis from the first day of tumor cell injection until death or occurrence of an event. (**E**) Tumors treated as above were analyzed by immunoblotting with indicated antibodies. (**F**) LG100754 reduced the blood glucose level and decrease lipid accumulation. (**Left**) Approximately 5 mg/kg LG100754 was injected intraperitoneally into mice. Blood glucose level was measured using glucose meter at indicated timepoint. (**Right**) Approximately 5 mg/kg LG100754 was injected intraperitoneally into mice. Total blood lipid was measured using lipid quantification kit. *: *p* < 0.05; **: *p* < 0.01.

To examine the therapeutic implications of our findings in vivo, we investigated the efficacy of the combined RXR agonist and lenalidomide in a preclinical model of lenalidomide-resistant human MM. We established a mouse xenograft model with MM1.R in SCID mice by implanting MM1.R cells subcutaneously in the flank of the mice. Once a tumor was established and palpable, the following were administered to the mice: PBS control, LG100754 (5 mg/kg, IP, three times a week), lenalidomide (10 mg/kg/day), or a combination of both (Figure 6A). The body weight was then measured. The mice were sacrificed if humane endpoints were reached or at the end of the experiments when the tumors were harvested. Notably, the combination of LG100754 and lenalidomide resulted in more effective tumor control as compared with LG100754 treatment alone, lenalidomide alone, or vehicle-alone (Figure 6A–C). Importantly, the combination treatment resulted in a significant prolongation of survival of the mice without any appreciable toxicity when their total body weight was used as a proxy (Figure 6B,D). In addition, the level of *CRBN* in the mouse tumors was increased in the LG100754 and lenalidomide combination-treated mice compared with those treated with lenalidomide alone, LG100754 alone, or PBS. Additionally, the expressions of cleaved caspases 3 and 9 were increased, while those of *IKZF1* and *IKZF3* were reduced in the LG100754 and lenalidomide combination group (Figure 6E). These data demonstrate a synergistic anti-myeloma effect when combining an *RXR* agonist with lenalidomide.

A previous study showed that LG100754 led to a reduction in glucose levels and improvement in insulin resistance in vivo [25]. To further characterize the effects of LG100754 on metabolic regulation, we performed a serum lipid analysis and fasting blood glucose testing at multiple timepoints after the LG100754 treatment in the animal models. C57Bl/6 mice were given 5 mg/kg LG100754 intraperitoneally, after which, their blood glucose and lipid levels were measured at various timepoints. Our data suggest that the LG100754 treatment resulted in an effective reduction of the blood glucose and lipid levels. The effects were observed starting at around one hour after the LG100754 treatment. The peak was reached at 6 h, while the effect lasted for 24 h (Figure 6F).

## 4. Discussion

*CRBN* is critical in immunomodulatory agent-mediated anti-myeloma activity and there are data suggesting that the level of *CRBN* correlates with drug sensitivity or resistance to IMiD treatment [53,54,55]. *CRBN* expression is a potentially useful marker to gauge the efficacy of IMiDs. Our previous research showed that *PPAR* agonists (fenofibrate, GW501516, and troglitazone) reduced the anti-myeloma effect of lenalidomide by downregulating the transcription activity of *CRBN* through DNA methylation and protein degradation. We found that *PPAR* agonists affected the *CRBN* promoter CpG island methylation pattern and caused hypermethylation in the *CRBN* promoter region. Moreover, *CRBN* was rapidly degraded upon exposure to *PPAR* agonists [43]. Although several mechanisms for this drug–drug interaction have been suggested, none has been proven.

Notably, the PPAR pathway reduces blood glucose and lipid levels. However, it is unknown whether these metabolic effects can be maintained while enhancing their *CRBN* expression. In this study, we explored the action of *RXR* heterodimer agonists (LG100754, bexarotene, AGN194204, and LG101506). We found that they enhanced MM sensitivity to lenalidomide in vitro and in vivo in a xenograft myeloma mouse model. Data from our CHiP and firefly/Renilla reporter analysis demonstrated that *RXR* agonists resulted in the attenuation of the binding effect of *PPAR* on the *CRBN* promoter region. This provides the first direct evidence that *RXR* agonists can increase *CRBN* expression. Moreover, our MSP analysis demonstrated that LG100754 and bexarotene affected the *CRBN* promoter CpG island methylation patterns, thereby favoring demethylation. Furthermore, we determined the dual role of LG100754 in T cell activation and exhaustion in Jurkat T cell lines and primary human T cells, suggesting their T cell protective and anti-exhaustion effects. Finally, our data demonstrate that the *RXR* agonist, LG100754, enhanced myeloma’s sensitivity to lenalidomide and reduced blood glucose and serum lipids in vivo; thus, administration of LG100754 may be an effective strategy to overcome drug resistance in the chemotherapeutic treatment of MM, while providing additional benefits for patients with comorbidities, such as diabetes and/or dyslipidemia. Our findings provide a strong justification for future clinical trial testing of the efficacy of *RXR* agonists in combination with IMiDs in patients with MM.

Our study is significant in several aspects. It provides the first evidence that RXR: PPAR heterodimer agonists are promising small molecule candidates that can upregulate *CRBN* expression and enhance the sensitivity of MM cells to IMiDs while maintaining glucose-lowering beneficial effects. These properties are significantly better than the PPAR agonists. Our previous studies showed that *PPAR* agonists reduced lenalidomide’s anti-myeloma activity [12,43]. These dual functions of *RXR* agonists are particularly relevant in the treatment of patients with MM. MM is predominately a disease of the elderly, a population at risk of having comorbidities, such as diabetes and dyslipidemia. Furthermore, they are more likely to require co-treatment with glucose- or lipid-lowering agents and immunomodulatory agents [56,57]. Dexamethasone is almost always incorporated in myeloma treatment regimens, including its combination with lenalidomide [58]; however, dexamethasone has been associated with increased blood glucose levels [59]. The combination of *RXR* agonists, such as LG100754 with lenalidomide and dexamethasone, could alleviate the hyperglycemia induced by dexamethasone, providing an additional advantage for this combination. Iberdomide and mezigdomide, which are novel *CRBN* E3 ligase modulators (CELMoDs), showed higher efficacy in lenalidomide-resistance MM patients in ongoing clinical trials. We expect similar or even more potent effects of RXR agonists when combined with these more specific *CRBN*-targeting drugs. Future research should focus on evaluating the combinations of *RXR* agonists with these agents. Importantly, the effects of *RXR* agonists in enhancing T cell activation while reducing T cell exhaustion provide additional benefits in the treatment of MM. The microenvironment and T cell immunity are crucial in maintaining the treatment response and in keeping patients under remission. This study provides proof-of-concept evidence for further exploration and development of new RXR:PPAR heterodimer agonists. These *RXR* modulators are potentially useful in treating myeloma patients who also have comorbidities, such as diabetes.

Several previous reports using isolated DNA or ligand-binding segments did not provide clear answers on how multiple domains act synergistically to modulate the receptor properties of PPAR:RXR heterodimers [60,61]. It has been shown that the balance of *PPAR* and *RXR* affects the heterodimerization of a specific RXR/PPAR complex [62]. A disproportion between the level of *PPAR* and the level of *RXR* resulted in less heterodimer formation capable of binding to the PPAR/RXR recognition site in the DNA. In addition, competition of *PPARs* for a limited amount of *RXR* to form a functional heterodimer may also impact the transcription of the target genes [63]. Our ChIP assay and *CRBN* promoter–luciferase reporter system demonstrated that RXR agonists reduced the binding of *PPARs* to the *CRBN* promoter and decreased the methylation status of the CpD islands of the *CRBN* promoter, thus reversing the repressor effects of *PPARs* on *CRBN* transcription. This observation was further supported by the results of our co-IP assay, which showed that *PPARs*, but not *RXR*, formed complexes with *EZH2*. These data support that *RXR* agonists act by affecting the binding and effect of *PPARs* on *CRBN* regulation. Interestingly, the *RXR* agonists retained or enhanced the other functions of *PPARs*, such as the regulation of glucose and lipid metabolism. Additional studies are needed in the future to determine the mechanisms resulting in these different effects of *RXR* agonists. CS018, a retinoid-like compound, was designed and synthesized based on the chemical structure of the *RXR* natural ligand [64]. Interestingly, CS018 facilitates the formation of RXR homodimers and the heterodimers of *RXR* with *PPARs*, but not *FXR* and *LXR*. Consistent with our data, CS018 induced the expression of *PPAR γ* target genes, *CD36*, and lipoprotein lipase (*LPL*) and significantly reduced animal blood glucose levels in vivo.

Bexarotene is an FDA-approved RXR agonist. It has undergone trials in multiple myeloma patients (NCT01504490) [65,66]. This study tested the safety and efficacy of a new combination of drugs of *PPARγ* agonists, such as CS7017 and bexarotene, on MM. Unfortunately, no conclusive benefits were found from the clinical trial. The combination strategy of bexarotene with IMiDs was never explored. Acquired drug resistance to IMiDs remains a major therapeutic issue in myeloma treatment. We believe that *RXR* agonists may have significant clinical applicability on the drug resistance of MM.

*RXR* agonists in general have a favorable side-effect profile. The effects of *RXR* agonists on lipid metabolism could vary depending on the individual agent. For instance, we found that LG100754 could lower the total lipid level in our animal study (Figure 6F). On the other hand, lipid abnormalities and hypertriglyceridemia are one of the common side-effects in patients treated with bexarotene. Bexarotene can elicit lipogenic side effects [67]. Other *RXR* agonists could elevate the “good” high-density lipoprotein and the “bad” triglycerides [68]. Although we did not observe significant weight changes when LG100754 was combined with lenalidomide in our mouse model (Figure 6B), it is possible that *RXR* agonists could increase the risks of bone marrow suppression, skin rashes, or thrombosis associated with the use of immunomodulatory agents. Additional studies are needed.

*RXR* and *PPAR* are ligand-activated transcription factors that form a heterodimer with each other or with other members of the *RXR* family [69]. The interaction occurs in the presence and absence of a *PPAR* ligand [70]. The RXR–PPAR heterodimers further recruit other cofactors before binding to PPRE at the promoter regions of *PPAR*-responsive genes, and these coactivators or corepressors are tightly involved in the regulation of target gene transcriptional activity [71,72]. T cell death-associated gene 51(TDAG51) was previously identified as a novel corepressor of *PPAR γ*-mediated transcriptional regulation. Overexpression of *TDAG51* reduced the differentiation of 3T3-L1 adipocyte cells. *TDAG51* physically interacted with *PPAR γ* in a ligand-independent manner, blocked *RXRα* recruitment to the RXRα–PPARγ heterodimer complex in adipogenesis, and negatively regulated the functions of *PPAR γ* by blocking [73].

Our finding on the effects of LG100754 on T cells is consistent with that of a previous study reporting that the LG100268, an RXR–PPARγ heterodimer agonist, has favorably modulated the immune microenvironment in preclinical breast cancer models [74,75]. Exposure to LG100268 increased the cytotoxic activity of CD8 T cells in tumors of MMTV-Neu mice (a model of HER2-positive breast cancer). This enhanced immunity of LG100268 was due to the combined effects of decreased infiltration of myeloid-derived suppressor cells and *CD206*-expressing macrophages, and an increased ratio of *CD8/CD4* and *CD25* T cells. LG100268 improved the response to an immune checkpoint blockade in breast cancer. Taken together, our studies demonstrate that the RXR:PPAR pathway plays important roles in immune regulation and disease pathogenesis, and that *RXR* agonists are novel agents with potential to modulate the functions of immune cells.

## 5. Conclusions

Our study demonstrates that *RXR* agonists significantly enhanced the sensitivity of the MM cells to lenalidomide in vitro and in vivo. *RXR* agonists upregulated *CRBN* transcription and attenuated the methylation status of the *CRBN* promoter. In addition, low concentrations of LG100754 protected T cells from exhaustion and improved the anti-tumor activity. Combining *RXR* agonists with lenalidomide resulted in more effective tumor control in the myeloma xenograft mouse model. The present study provides new information regarding the *PPAR*–*RXR*–*CRBN* interaction, which may be useful in guiding future studies and treatment. Our study provides novel targets for improving the efficacy of immunomodulatory agents for patients with MM, and it is particularly relevant to myeloma patients who have co-existing diabetes or dyslipidemia.

## Data Availability

The data presented in this study are available on request from the corresponding author.

## Supplementary Materials

The following supporting information can be downloaded at: https://www.mdpi.com/article/10.3390/cells12151993/s1, Figure S1: MM1.R, U266 were treated with LG100754, bexarotene, AGN194204, and LG101506 for 48 h at indicated concentration and cell viability was measured by MTS assay. Figure S2: Synergistic suppression of MM cell proliferation using lenalidomide and LG100754. Figure S3: Bexarotene attenuates the binding effect of PPAR γ on the CRBN promoter area. U266 and MM1.R were transfected with CRBN/PGL3 firefly luciferase reported vector construct. Transfected cells were then co-treated with PPAR agonist (troglitazone) and/or RXR agonist bexarotene for 48 h, and luciferase activity was measured. Results are presented as mean ± SD from at least three separate experiments. *: *p* < 0.05; **: *p* < 0.01; NS: not statistically significant. Figure S4: Bar graphs show qRT-PCR data using immunoprecipitated DNA obtained from ChIP with anti-CRBN or anti-IgG (negative control) antibodies; error bars represent SD. *: *p* < 0.05; **: *p*<0.01; NS: not statistically significant. Figure S5: LG100754 regulates CRBN transcription activity via promotion of heterodimer formation between PPAR and CRBN. Figure S6: (A) CpG island prediction using MethPrimer software; “a” represents the methylated regions; (B) Predicted sequences of the CRBN design using MethPrimer; methylated nucleotides are indicated with “+”, unmethylated nucleotides with “:”, and other nucleotides with ‘|.’. Figure S7: Co-IP assay between RXR with EZH2 was conducted with the indicated antibody in U266 and MM1.R. Figure S8: Raw data for Figure 2A. Figure S9: Raw data for Figure 2C. Figure S10: Raw data for Figure 4 and Figure 5.
