# Supplementary material for: RXR Agonists Enhance Lenalidomide Anti-Myeloma Activity and T Cell Functions while Retaining Glucose-Lowering Effect
Source: Cells. 2023 Aug 3;12(15):1993. doi: 10.3390/cells12151993 (PMC10417536; doi:10.3390/cells12151993)
Supplement: Supplementary file 1 [file cells-12-01993-s001.zip › cells-2487517-supplementary.pdf]

RXR agonists enhance lenalidomide anti-myeloma activity and T cell functions while retaining glucose lowering effect

Jian Wu[1], Xiaobei Wang[1], Min Zhang[1], Parker Mathews[1], and Yubin Kang[1*]

[1]Division of Hematologic Malignancies and Cellular Therapy, Department of Medicine, Duke University Medical Center, Durham, North Carolina, USA.

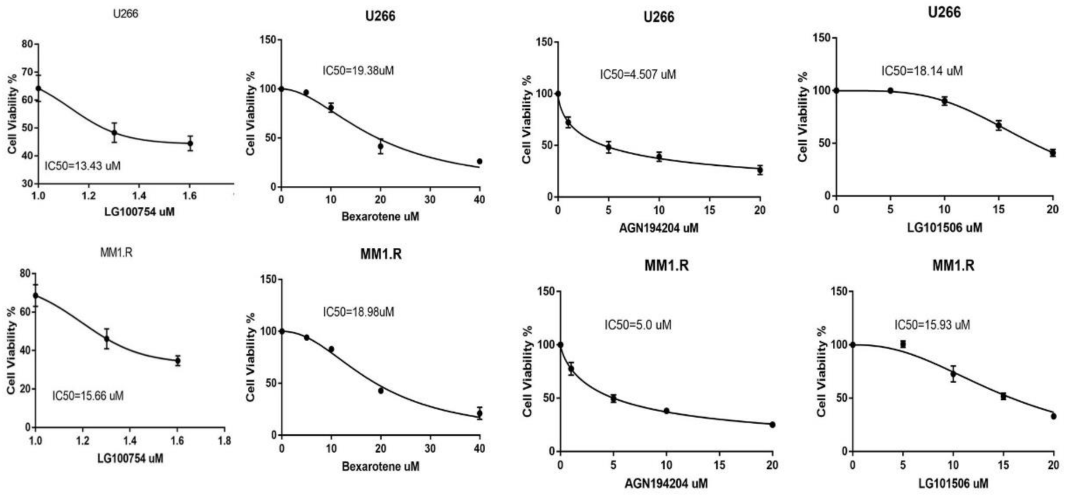

Figure S1. MM1.R, U266 were treated with LG100754, Bexarotene, AGN194204, and LG101506 for 48h at indicated concentration and cell viability was measured by MTS assay.

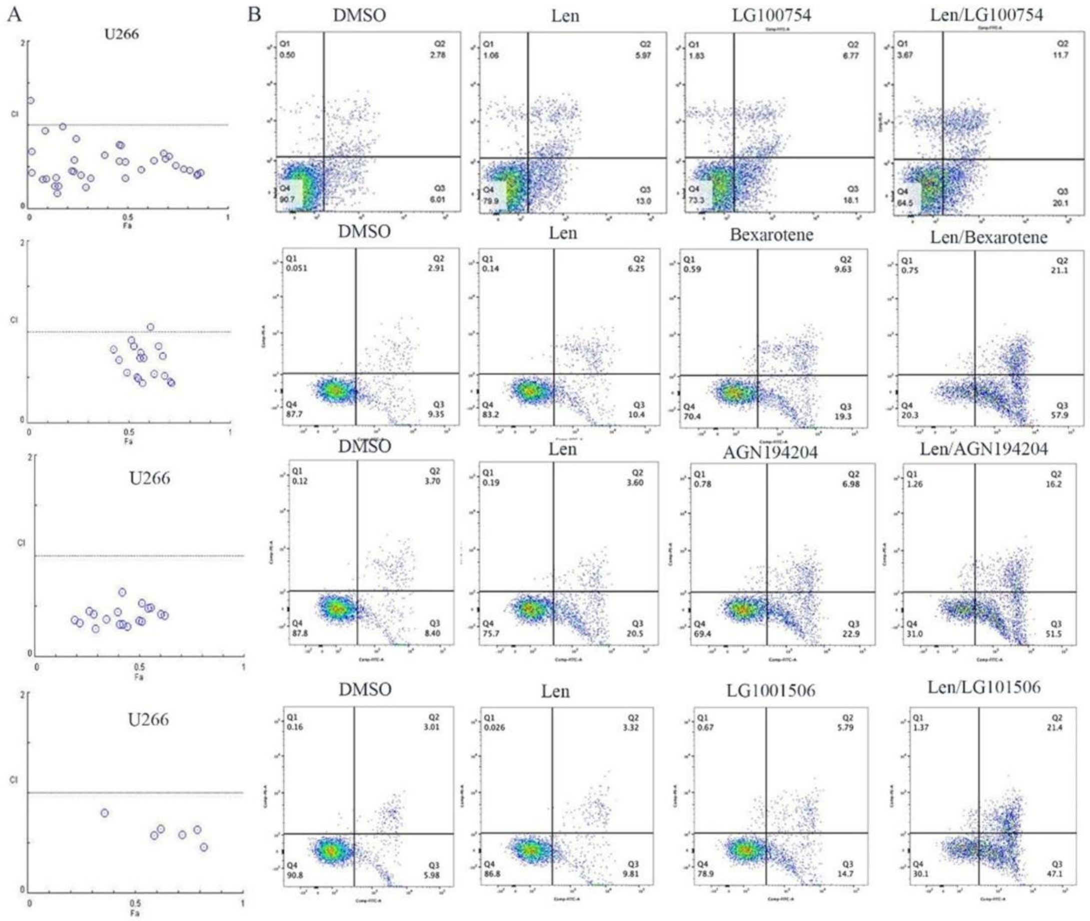

Figure S2. Synergistic suppression of MM cell proliferation using lenalidomide and LG100754. (A) U266 were treated with multiple concentrations for 48h, and combination index (CI) value identified using CompuSyn software. (B) U266 were treated with the above concentrations of Lenalidomide and RXR agonists for 48h, cell apoptosis was measured using Annexin V/PI staining assay.

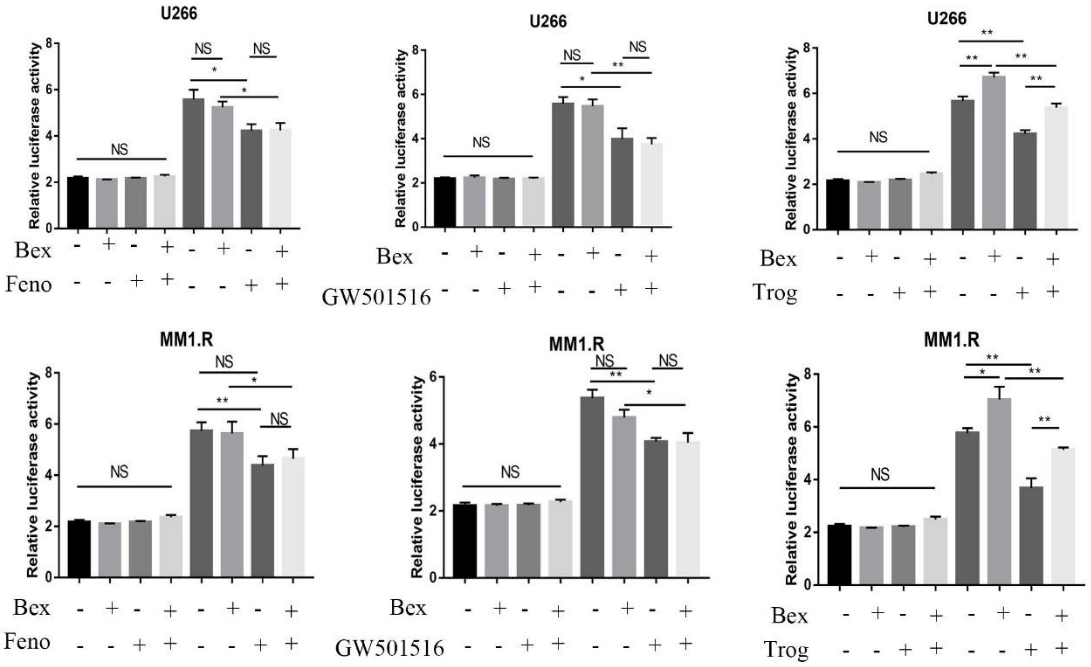

Figure S3. Bexarotene attenuates the binding effect of PPAR γ on the CRBN promoter area. U266 and MM1.R were transfected with CRBN/PGL3 firefly luciferase reported vector construct. Transfected cells were then co-treated with PPAR agonist (troglitazone) and/or RXR agonist Bexarotene for 48h, and luciferase activity was measured. Results are presented as mean ± SD from at least three separate experiments. *: p<0.05; **: p<0.01; NS: not statistically significant.

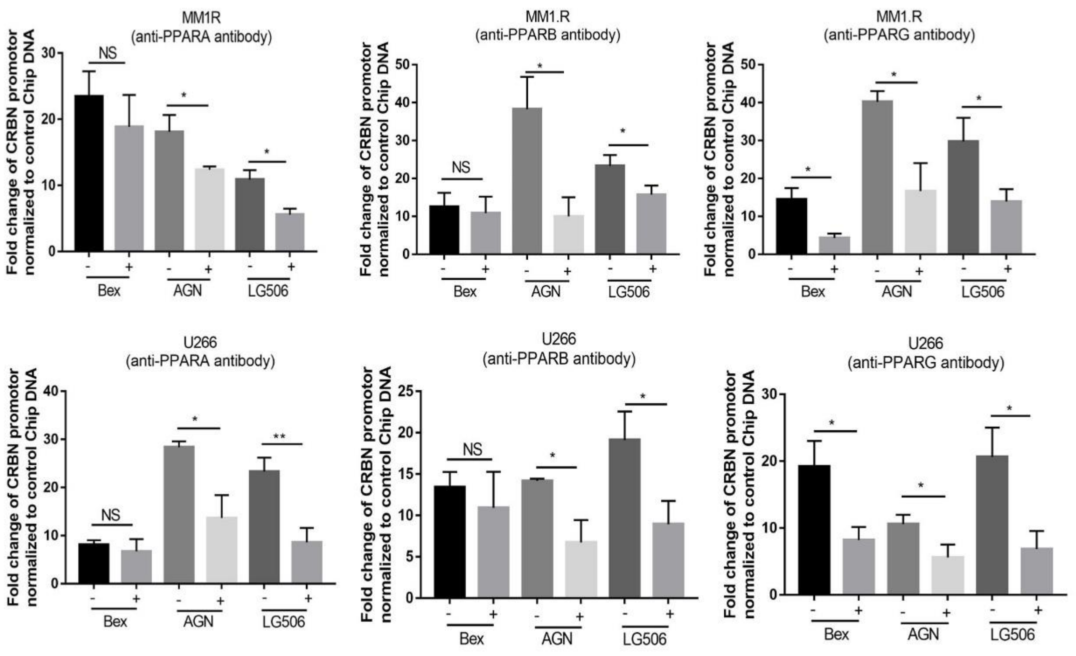

Figure S4. Bar graphs shows qRT-PCR data using immunoprecipitated DNA obtained from ChiP with anti-CRBN or anti-IgG (negative control) antibodies, error bars represent SD. *: p<0.05; **: p<0.01; NS: not statistically significant.

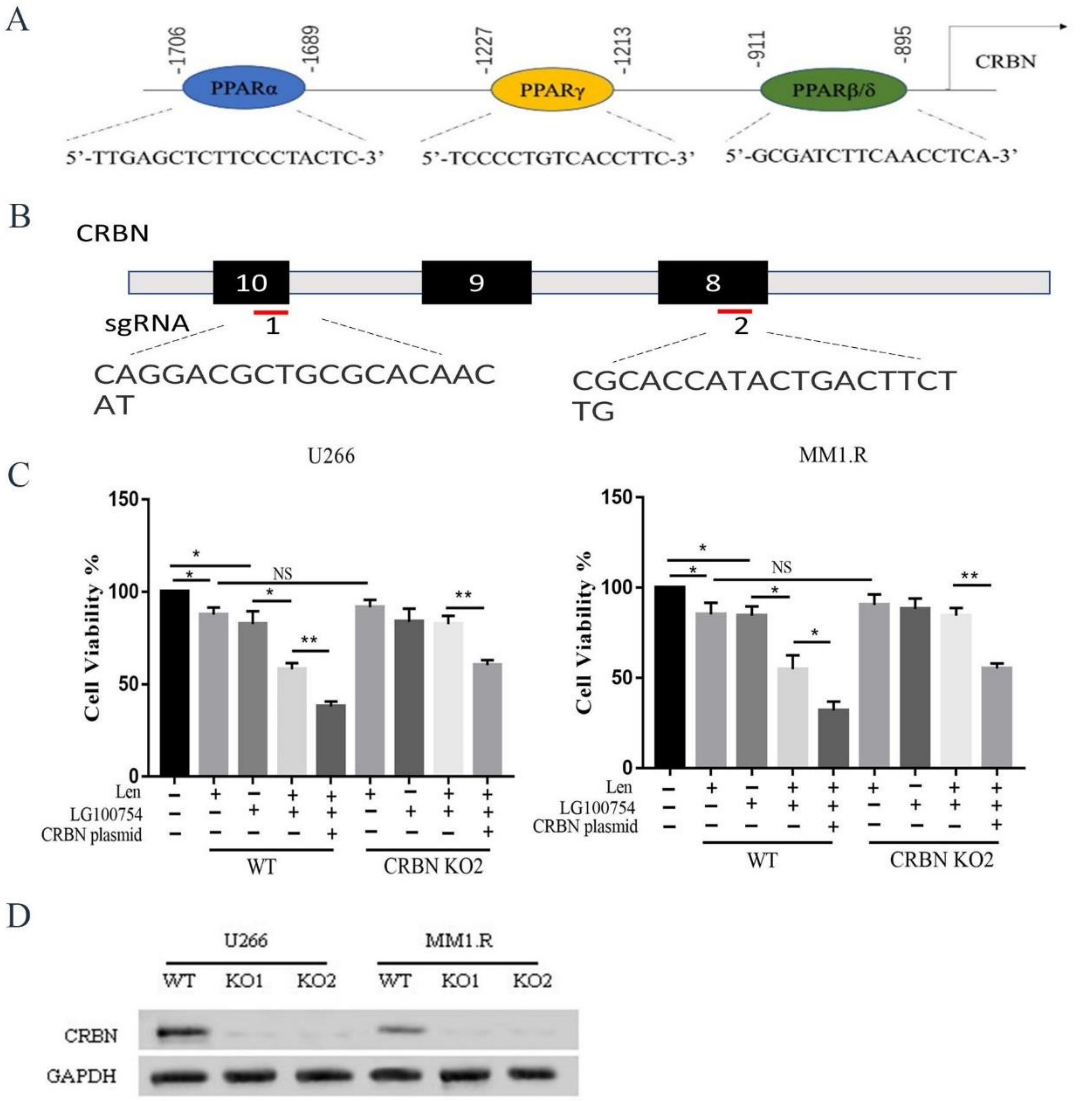

Figure S5. LG100754 regulates CRBN transcription activity via promotion of heterodimer formation between PPAR and CRBN. (A) JASPAR databases predict binding sites of PPAR to CRBN promoter region. (B) sgRNA sequence used to knock out CRBN in MM1.R and U266. (C, D) MM1.R and U266 cells were transduced with CRBN specific CRISP/cas9 knock out plasmid for 24h and then treated with lenalidomide or LG100754 alone or in combination for additional 48h. Cell viability was measured by MTT assay. Results are presented as mean ± SD from at least three separate experiments. *: $p<0.05$; **: $p<0.01$. Protein lysate was subjected to western blot with indicated antibodies.

A

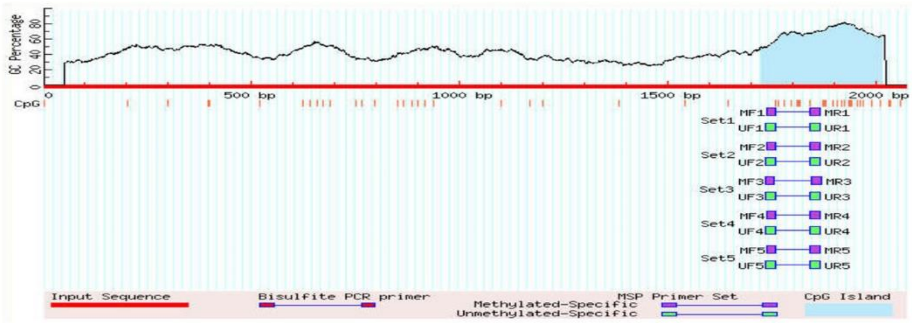

B

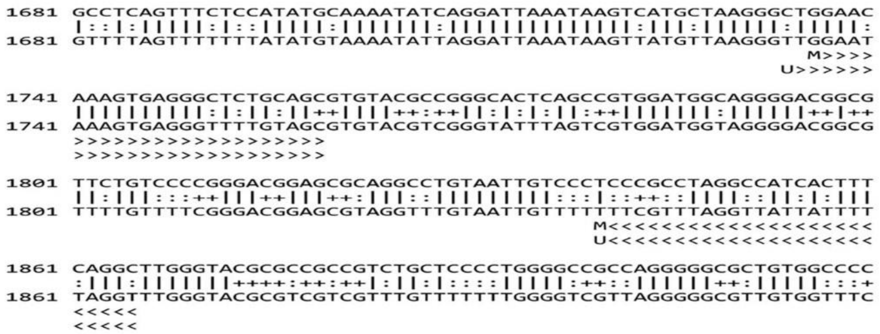

Figure S6. (A) CpG island prediction using MethPrimer software, “a” represent the methylated regions; (B) Predicted sequences of the CRBN design using MethPrimer, Methylated nucleotides are indicated with “+”, unmethylated nucleotides with “:” and other nucleotides with ‘|.’.

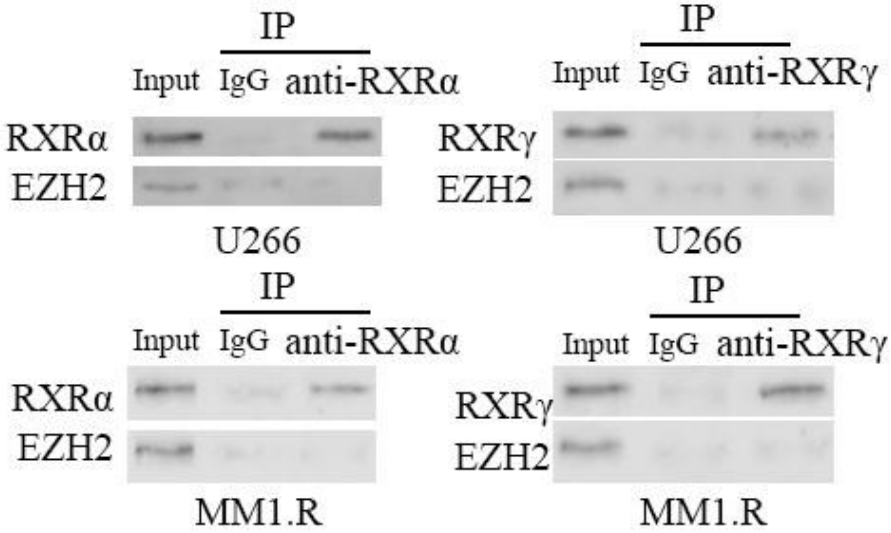

Figure S7. Co-IP assay between RXR with EZH2 was conducted with the indicated antibody in U266 and MM1.R.

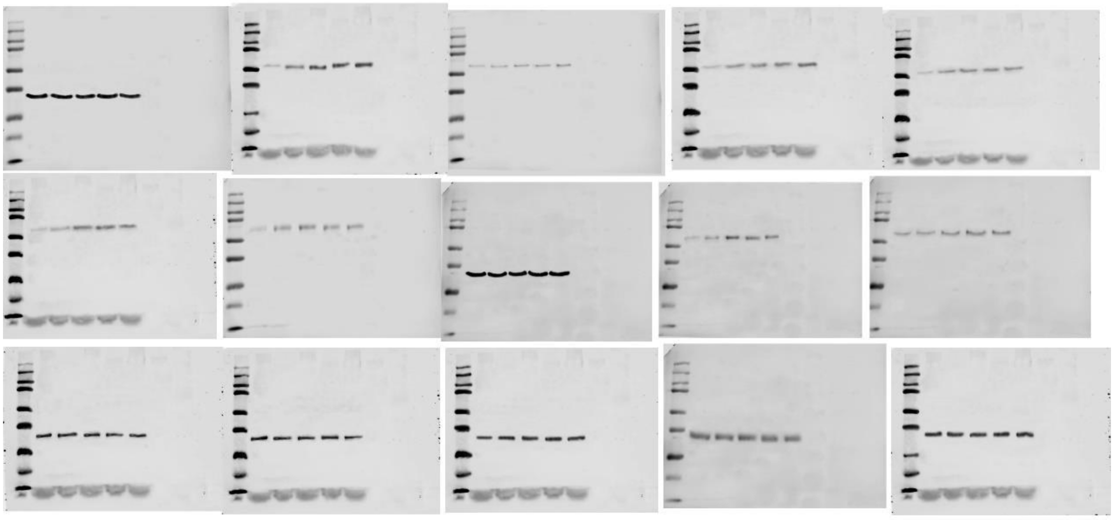

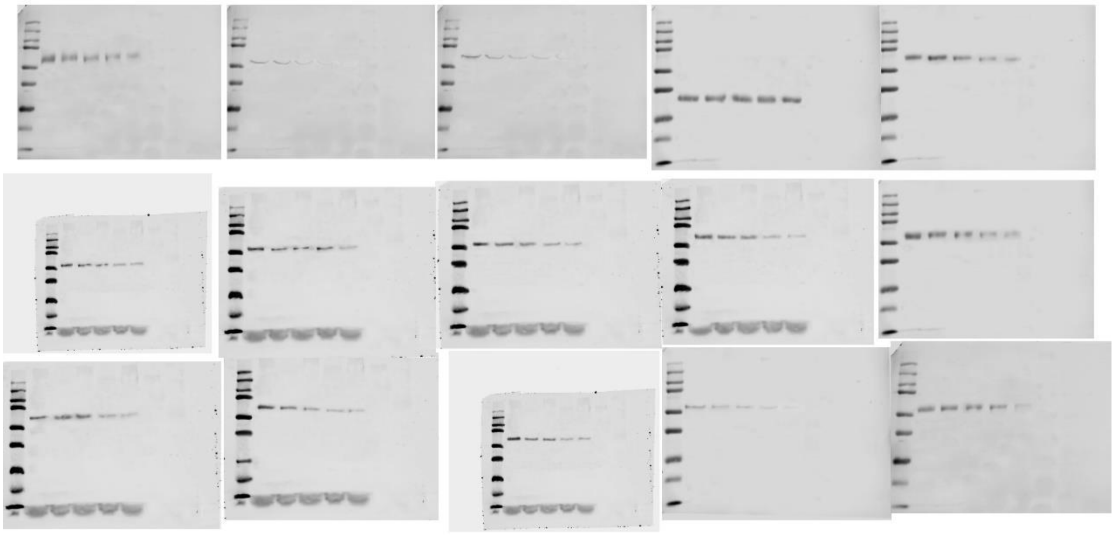

Figure S8. Raw data for Figure 2A.

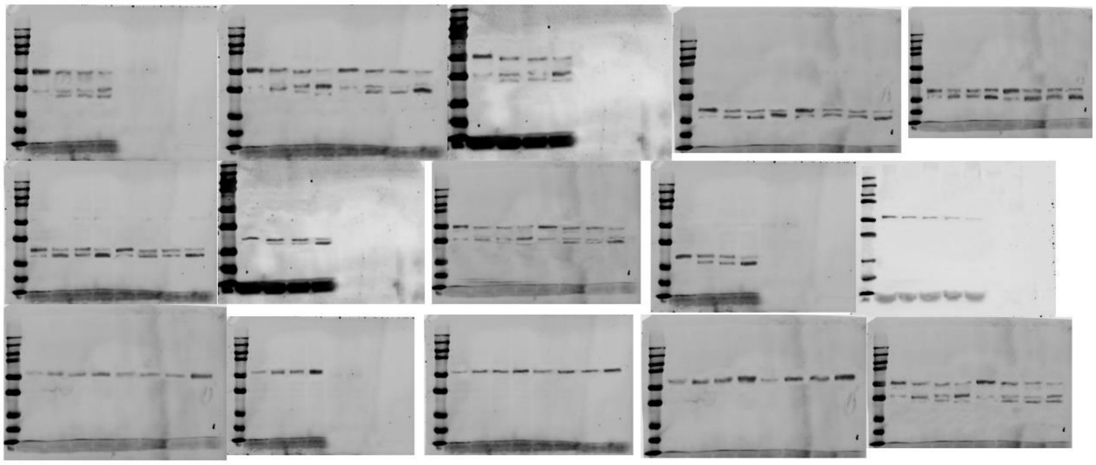

Figure S9 Raw data of figure 2C

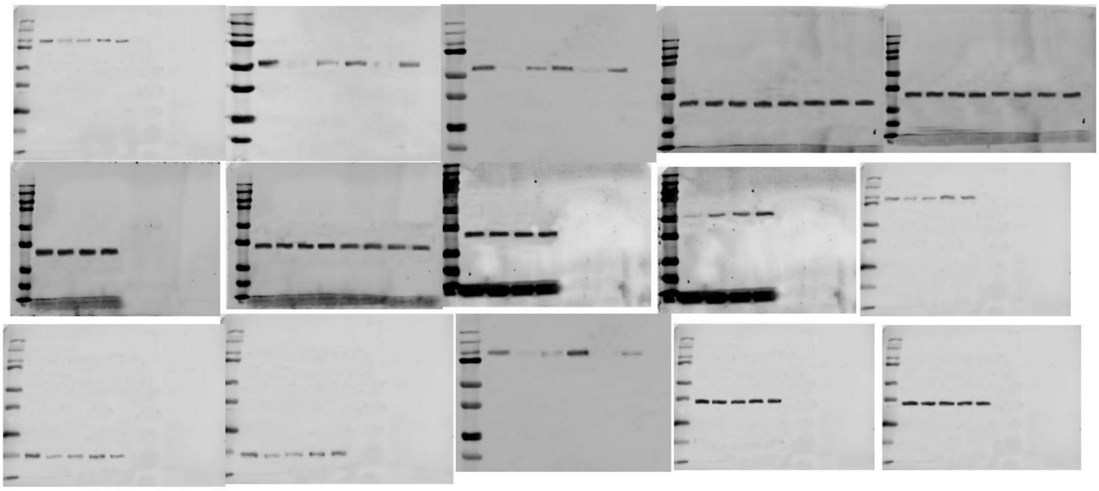

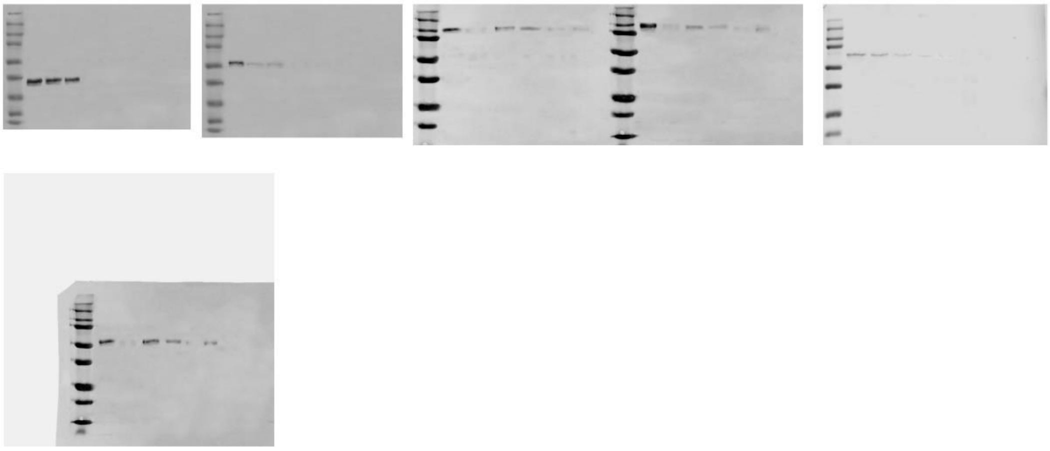

Figure S10. Raw data for Figure 4 and Figure 5.
